# Fullerene C60 Protects Against Intestinal Injury from Deoxynivalenol Toxicity by Improving Antioxidant Capacity

**DOI:** 10.3390/life11060491

**Published:** 2021-05-27

**Authors:** Simeng Liao, Guang Liu, Bie Tan, Ming Qi, Jianjun Li, Xiaoqing Li, Changfeng Zhu, Jiamei Huang, Yulong Yin, Yulong Tang

**Affiliations:** 1 Laboratory of Animal Nutritional Physiology and Metabolic Process, Key Laboratory of Agro-Ecological Processes in Subtropical Region, National Engineering Laboratory for Pollution Control and Waste Utilization in Livestock and Poultry Production, Institute of Subtropical Agriculture, Chinese Academy of Sciences, Changsha 410125, China; liaosaimeng16@mails.ucas.ac.cn (S.L.); lg@stu.hunau.edu.cn (G.L.); Qiming16@mails.ucas.ac.cn (M.Q.); jianjunli@isa.ac.cn (J.L.); 2 Institute of Subtropical Agriculture, Chinese Academy of Sciences, Beijing 100049, China; 3 College of Animal Science and Technology, Hunan Agricultural University, Changsha 410128, China; bietan@isa.ac.cn; 4 Xiamen Funano New Material Technology Company, LTD, Xiamen 361005, China; czj123@stu.hunau.edu.cn (X.L.); Zhaandong18@mails.ucas.ac.cn (C.Z.); tangshengguo1983@hunau.edu.cn (J.H.)

**Keywords:** deoxynivalenol, fullerene C60, inflammatory, oxidative stress, intestinal

## Abstract

Oxidative stress is involved in a wide variety of pathologies, and fullerene has been shown to have an antioxidant ability. Mycotoxins exert toxic effects through induction of excessive reactive oxygen species (ROS). Here, we evaluated water-soluble fullerene C60 for its anti-mycotoxin and antioxidant effects in vitro and in vivo. Intestinal epithelial cells were cultured with fullerene during deoxynivalenol (DON) exposure. The results revealed that fullerene C60 significantly promoted cell viability, decreased apoptosis and necrotic cell number, and significantly reduced intracellular ROS levels during DON exposure (*p* < 0.05). To investigate the role of fullerene in antioxidant capacity in vivo further, 40 three-week-old male C57BL/6 mice were randomly divided into four groups. There were no significant differences between the control and fullerene groups (*p* > 0.05). In mice exposed to DON, supplementation with fullerene C60 significantly improved growth performance, and enhanced the total antioxidant status and the activities of SOD and GPX in the intestine and liver (*p* < 0.05). In addition, fullerene C60 supplementation improved intestinal morphology, as indicated by a higher villus height and tight junction protein expression (*p* < 0.05). Furthermore, fullerene supplementation decreased serum concentrations of inflammatory cytokine and lipopolysaccharide (LPS; a penetrability marker) compared to the DON-challenged group (*p* < 0.05). The current study suggests that fullerene C60 improves intestinal antioxidant status against DON-induced oxidative stress in vitro and in vivo.

## 1. Introduction

The use of fullerene C60, as a new therapeutic approach, is being intensely developed by exploring cellular signaling pathways. In recent years, fullerene C60 and its derivatives have exhibited various beneficial bioactivities, including anti-bacterial [1], anti-viral [2], anti-inflammatory [3], neuroprotective [4], autophagic [5], antitumor [6], and anti-aging [7]. Previous research has found that fullerene C60 specifically binds to and activates the hippocampal Ca2+ signaling protein (CaMKIIα) to increase learning and memory [8]. Moreover, the use of fullerene C60 for tumor treatment has been reported, where it enhances the antitumor effect by mediating CaMKIIα activity [6]. Several other studies have documented remarkable antitumor effects of fullerenes via a variety of mechanisms involving immunomodulation, autophagy modulation, and oxidative stress [5,9,10].

Excess free radicals and oxidative stress are generally known to be detrimental to human health [11], and several diseases have been associated with increased levels of oxidative stress, including cancer, Alzheimer’s disease, Parkinson’s disease, inflammatory disorders, and asthma [6,12,13]. The antioxidant activities of fullerene C60 have been demonstrated in previous reports, showing that the antioxidant capacity of C60 is 125 times that of vitamin C, and the mechanism may be the REDOX reaction between ROS and fullerene molecules through direct electron transfer, such that ROS is finally decomposed [14,15]. It has been reported that fullerene-C60-treated rats have high resistance to CCl4, the toxicity of which is mediated by ROS production [16]. Studies have shown that another mechanism of fullerene oxidation resistance is based on the mild decoupling of the respiratory chain and phosphorylation in the mitochondrial inner membrane [14].

The induction of ROS is considered a common mechanism of various toxicities of mycotoxins [17,18]. Monosporophene toxoids can interfere with the normal functioning of mitochondria and produce ROS and other free radicals. These substances can cause lipid peroxidation and change the antioxidant system of cells, reducing the activity of antioxidant enzymes, such as glutathione transferase (GST), superoxide dismutase (SOD), and catalase (CAT) [19]. This process leads to DNA damage, increased ROS production, and lipid peroxidation, followed by activation of inflammatory signaling pathways such as MAPK, JAK/STAT, and NF-κB, causing cell apoptosis and necrosis [20]. The antioxidant capacity of antioxidants is essential for the detoxification of monosporophenes. For example, the traditional antioxidants (vitamins A, C, and E) show some resistance to protein and lipid peroxidation induced by deoxynivalenol (DON) [21,22], an antimicrobial peptide, and can improve the feed conversion rate, immune function, and antioxidant ability, and can reduce the organ damage caused by DON [23]. In addition, some plant extracts, including carob acid (LA), polyphenolic epigallocatechin gallic acid (EGCG), and papaya seed mucus, have noticeable anti-oxidative stress effects caused by monosporophenes [24]. Therefore, it is imperative to seek better antioxidants. However, the extent to which research on nanoscale C60 can affect the toxicities of mycotoxins is unclear. This report evaluated nano-fullerene C60 for anti-mycotoxin and antioxidant effects in vitro and in vivo. In parallel, our research about the biological antioxidant capacity of nano-C60 will boost fullerene C60, as a powerful antioxidant, contribution to human and animal health.

## 2. Materials and Methods

### 2.1. Materials and Reactions

Immortalized wild-type IPEC-J2 and HCT8 cells from our laboratory (Institute of Subtropical Agriculture, Changsha) were used. Fullerene C60 was purchased from the Xiamen Funano New Material Technology Company. DCFH-DA (35845) and DON were purchased from Sigma (St. Louis, MO, USA). Primers used for RT-PCR were purchased from Sangon (Shanghai, China). All cell culture reagents were purchased from Gibco (Paisley, Scotland, UK) unless otherwise noted. 

### 2.2. Cell Culture

All cells were cultured in Dulbecco’s modified eagle medium (DMEM), and supplemented with 10% fetal bovine serum (FBS), 100 IU/mL penicillin G, and 100 g/mL streptomycin at concentrations of 37 ℃ and 5% carbon dioxide (CO_2_). The cells were incubated in a medium containing either DON (1.5 μg/mL) or fullerene C60 (1000 μg/mL). Microcapsule powder is used to encapsulate fullerene to increase its solubility and promote its mixing in feed.

### 2.3. Cell Proliferation Measurement

The cell proliferation was evaluated by a cell counting kit-8 (CCK-8) assay (Yeasen Biotechnology (Shanghai) Co., Ltd., Shanghai, China) according to the manufacturer’s instructions. IPEC-J2 cells were seeded in a 96-well plate at a density of 1×10^4^ cells/well and were incubated at 37 °C in a CO2 incubator. Absorbance at 490 nm was measured using a Microplate Reader (BioTEK, Winooski, VT, USA) at 450 nm. 

### 2.4. ROS and Apoptosis Measurement

The ROS levels were quantified by staining with dichloro-dihydro-fluorescein diacetate (DCFH-DA; Sigma-Aldrich, St. Louis, MO, USA). An equal number of cells were collected after treatment with DON and stained with DCFH-DA for 30 min at 37 °C, after which they were washed with PBS and analyzed using the flow cytometer. For each sample, 15,000–20,000 events were recorded. Cells were gated using the forward (FSC) and side (SSC) scatter before assessing for fluorescence. Cells with a fluorescence signal above the background, indicating positive as ROS generation is positive, were detected in the FL1channel (excitation (Ex) at 488 nm and emission (Em) at 530 nm).

To determine cell apoptosis, cells were treated with 10 μL Annexin V-FITC (20 μg/ mL) and 5 μL PI (50 μg/ mL) for 20 min. After washing with PBS three times, cells were detected by fluorescence microscopy.

### 2.5. Transcriptome Sequencing (RNA-Seq)

The adherent cell-culture medium was aspirated, and cells were washed once with PBS. The cells were digested with trypsin and collected in a 1.5 mL centrifuge tube, centrifuged at 1000 rpm for 5 min at 4 °C, then discarded the supernatant and frozen in liquid nitrogen. Transcriptome sequencing (RNA-seq) was conducted by the Shenzhen BGI Technology Company. RPKM, Reads Per Kilobase of exon model per Million mapped reads, is defined in this way [25].

### 2.6. Animals and Experimental Design

A total of 40 male C57BL/6 mice aged 3 weeks (average initial body weight (BW) is 13.13 ± 0.69 g) were divided into four groups. All mice had similar genetic backgrounds. Groups were divided as follows: control of basal diet and clean water (excluding DON), mice with basal diet and DON water (DON, 2 mg/L), mice with C60-contaminated diet and DON water, mice with C60-contaminated diet and clean water. From day 3 to day 14, C60 and DON were administered to mice. Other parameters include: darkness and light for 12 h each day, 23 ± 2 ℃; mice belonging to the same group were in the same cage, with the same cafeteria feeding and drinking water. Every three days, the bodyweight of all the mice was measured. The basal diet was formulated to meet the nutrient requirements conforming to the China General Quality Standards for Animal Feed (GB14924.1-2001) and has been described in a previous study [26].

### 2.7. Sample Collection and Preparation

On day 15, after fasting overnight, the mice were anesthetized, and blood was collected through the eye socket, bathed at 37 °C for 2 h, stood at 4 °C for 12 h, and centrifuged at 4000 r/min for 15 min, and the serum was collected into a 1.5 mL centrifuge tube and frozen at −80 ° C for further determination. After blood collection, liver, spleen, intestine, and feces samples were collected. The intact liver and spleen were weighed, and the relative organ index was calculated, as previously [26].

### 2.8. Detection of Inflammatory Cytokines, Immune Factors, and LPS Marker

Serum concentrations of immunoglobulin (Ig) G, IgM, IgA tumor necrosis factor (TNF)-α, interleukin (IL)-1β, IL-6, IL-8, IL-10, IL-12, transforming growth factor (TGF)-β, and lipopolysaccharide (LPS) marker were determined using commercial ELISA kits (Meimian Co. Wuhan, China), following the manufacturer’s instructions, and the resulting solutions were read on a microplate reader at 450 nm t [27]. 

### 2.9. Real-Time Quantitative Reverse Transcriptase PCR

A quantitative real-time polymerase chain reaction (RT-PCR) was conducted following a previous study [27]. In brief, total RNA was first extracted using the Trizol reagent (Invitrogen, Thermo Fisher Scientific, Waltham, MA, USA). RNA was purified and extracted using the RNeasy Mini Kit (Takara Bio Inc., Shiga, Japan). Secondly, deoxyribonuclease was used to remove DNA pollutants (Takara, Shuzo, Kyoto, Japan). The quality and quantity of the total RNA were determined using ultraviolet spectroscopy (Nanodrop 2000 Spectrophotometer, Thermo Scientific, Courtaboeuf, Villebon-sur-Yvette, France). The cDNA library was constructed using the cDNA Synthesis SuperMix kit (Transgen, Beijing, China), and diluted according to the instructions. Amplification conditions were performed as previously [27], and the primers used in the experiment are shown in Table 1.

### 2.10. Tissues Antioxidative Capacity

The levels of malondialdehyde (MDA), catalase (CAT), glutathione peroxidase (GSH-Px), and superoxide dismutase (SOD), and total antioxidant capacity (T-AOC) levels in liver, spleen, and ileum were determined according to manufacturer instructions using commercial ELISA kits (Meimian Co. Wuhan, China). The resultant solutions were read on a microplate reader. 

### 2.11. Intestinal Morphology

Intestinal morphology was analyzed, as described previously [27]. Crypt depth and villous height were measured and counted with computer-assisted microscopy (Micro metrics TM; Nikon ECLIPSE E200, Tokyo, Japan). 

### 2.12. Statistical Analysis

Data were analyzed by analyzing variance, using the SPSS 20.0 (SPSS Inc., Chicago, IL, USA), of the General Linear Models procedure. Significant differences between means and *p*-values were determined using Tukey’s multiple comparison tests. Results on the column chart were expressed as the mean ± standard deviation (SD). Differences were declared as significant at *p <* 0.05, and trends toward significance are discussed at 0.05 ≤ *p <* 0.10 

## 3. Results

### 3.1. Cell Proliferation, ROS Level, and Cell Apoptosis

**Figure 1:**
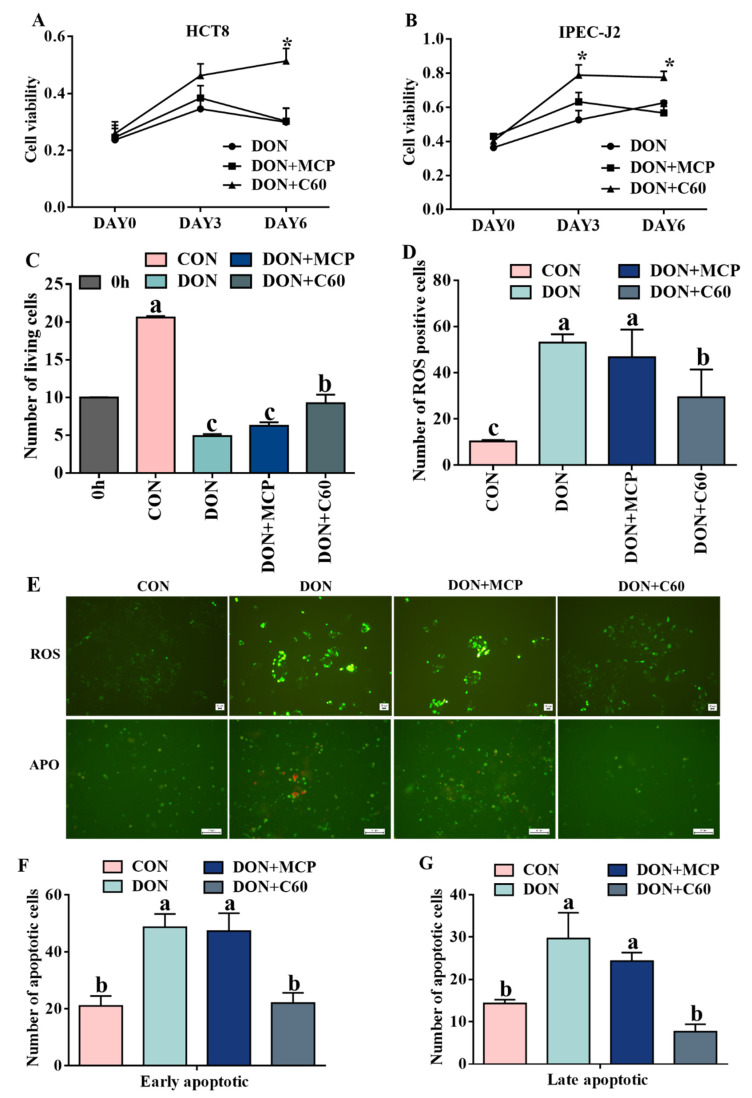
Cell proliferation, ROS level, and cell apoptosis. (**A**) Cell proliferation measurement of HCT8, DON, 1 ug/mL, C60, 1000 ug/mL. (**B**) Cell proliferation measurement of IPEC-J2, DON, 1.5 ug/mL, C60, 1000 ug/mL. (**C**) The count of living cells of IPEC-J2 after 48 h of treatment, 10^5^. (**D**) ROS (green fluorescence) and apoptosis (green represents early apoptosis and red-dish-brown represents late apoptosis or necrosis), measurement after 24 h of treatment. (**E**) The number of apoptosis cells. (**F**) and (**G**) The number of ROS-positive cells. The statistical assay used one-way ANOVA (*n* = 3, 3-wells of cells). Data are expressed as means ± SD, four models chosen; (**A**,**B**): * *p* < 0.05 vs. control. (**D**–**F**), ^a,b,c^ values within a row with different superscripts differ significantly (*p* < 0.05).

The cell counting Kit-8 (CCK-8) results (Figure 1A,B) showed that DON treatment inhibited cell proliferation and caused cell death in HCT-8 at day 6 (*p <* 0.010) and IPEC-J2 at day 3 (*p* = 0.036) and 6 (*p <* 0.010). The addition of fullerene C60 significantly promoted cell viability (Figure 1C) and decreased cell death (*p <* 0.010). 

Fluorescence analysis showed that the addition of fullerene C60 significantly reduced the ROS production level and the number of apoptotic and necrotic cells compared with DON treatment (*p <* 0.01, Figure 1D–F). There were no significant differences between the DON and microcapsule powder (MCP) groups that were used to encapsulate C60 (*p* = 0.061).

### 3.2. Cell Antioxidant Related Genes and Antioxidant Enzymes

**Figure 2:**
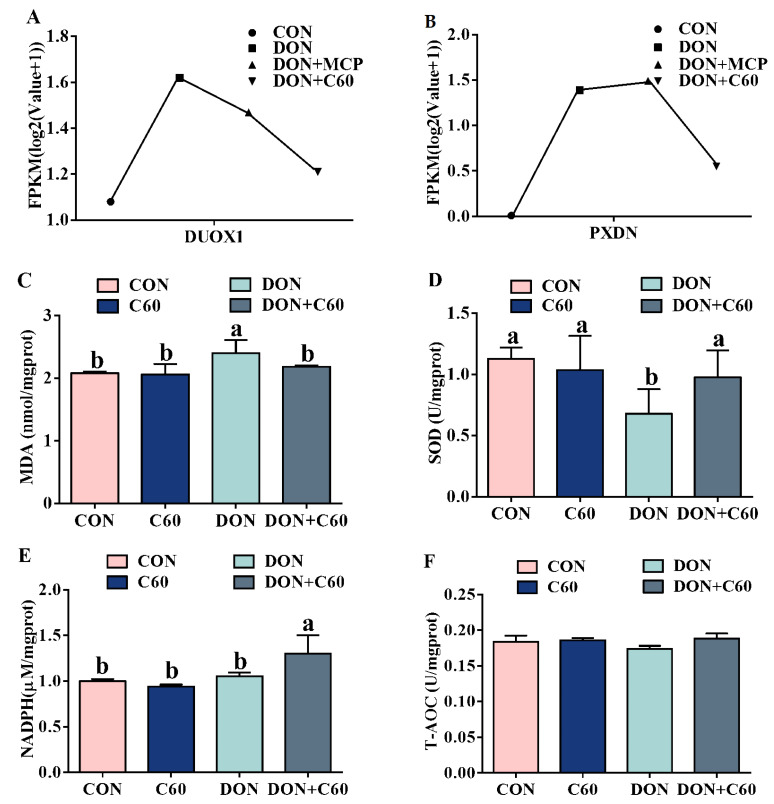
Cell antioxidant-related genes and antioxidant enzymes. (**A**) Sample expression of dual oxidase 1 (DUOX1); RPKM, Reads Per Kilobase of exon model per Million mapped reads, is defined in this way (Mortazavi et al., 2008). (**B**) Sample expression of peroxidase (PXDN). (**C**) The level of malondialdehyde (MDA) in IPEC-J2. (**D**) The level of superoxide dismutase (SOD) in IPEC-J2. (**E**) The level of nicotinamide adenine dinucleotide phosphate (NADPH) in IPEC-J2. (**F**) The level of total oxidation resistance (T-AOC) in IPEC-J2. The statistical assay used one-way ANOVA (*n* = 3, 3-wells of cells). Data are expressed as means ± SD, four models chosen; ^a,b^ values within a row with different superscripts differ significantly (*p* < 0.05).

The four samples were used for transcriptomic analyses by RNA-seq, by comparing antioxidant related genes; two genes, dual oxidase 1 (DUOX1) and peroxidase (PXDN), showed a significant increase in the DON group, while the addition of C60 reduced expression of these genes, suggesting the DON+C60 group can alleviate the oxidative stress (Figure 2). The cellular antioxidant index indicated a concordant expression pattern of oxidative stress between RNA-seq and enzymatic activity (Figure 2).

### 3.3. Growth Performance and Visceral Index

**Figure 3:**
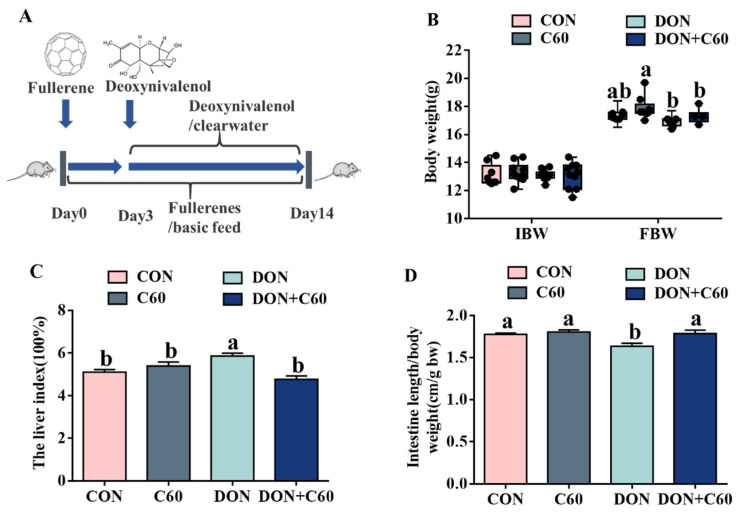
Growth performance and Visceral index. (**A**) Schematic of the timeline of the experiment. (**B**) Initial and final body weight of C57BL mice. (**C**) The liver index, the ratio of liver weight to body weight. (**D**) The ratio of intestine length to body weight. The statistical assay used one-way ANOVA. Data are expressed as means ± SD (*n* = 8), four models chosen; ^a,b^ values within a row with different superscripts differ significantly (*p* < 0.05).

Compared with the control group, the mice exposed to DON showed significantly reduced final body weight and intestinal length (Figure 3), as well as a higher liver index (*p =* 0.038), whereas dietary-supplemented fullerene C60 alleviated the detrimental effects owing to DON toxicity, matching growth performance and intestinal and liver indices measured in the control group. For mice supplemented with only fullerene C60, a higher final body weight relative to that of the control group was achieved (*p =* 0.032), but no response was found in other indices.

### 3.4. Serum Immunoglobulin and Inflammatory Factors

**Figure 4:**
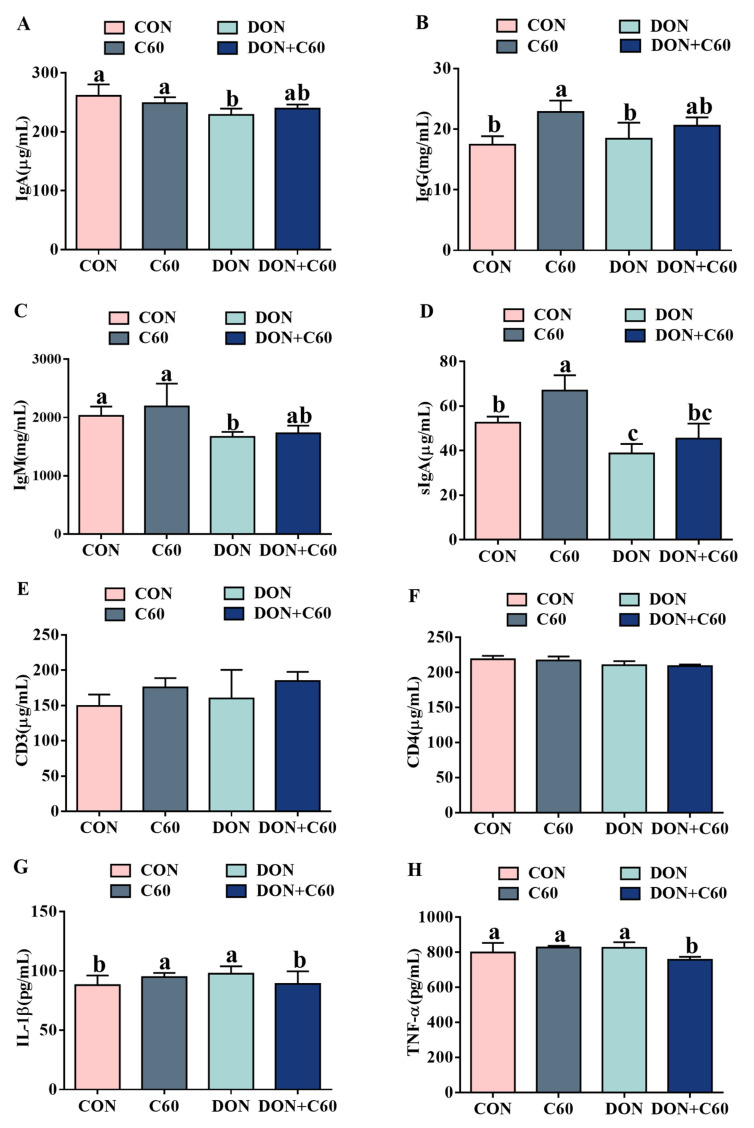
Serum immunoglobulin and inflammatory factors. (**A**) Immunoglobulin A (IgA). (**B**) Immunoglobulin G (IgG). (**C**) Immunoglobulin M (IgM). (**D**) Secretory immunoglobulin A (sIgA). (**E**) Complement 3 (C3) in blood serum. (**F**) Complement 4 (C4) in blood serum. (**G**) Interleukin -1β(IL-1β) in serum. (**H**) Tumor necrosis factor-α (TNF-α) in serum. The statistical assay used one-way ANOVA. Data are expressed as means ± SD (*n* = 8), four models chosen; ^a,b,c^ values within a row with different superscripts differ significantly (*p* < 0.05).

Compared with the control group, the mice treated with DON showed significantly (*p <* 0.05) lower concentrations of IgA, IgM, and SIgA (Figure 4A–D), whereas the addition of fullerene C60 promoted immune function, with higher concentrations of IgG and SIgA relative to the those of the DON group (Figure 4B,D) (*p <* 0.05). No difference of CD3 and CD4 among 4 groups (Figure 4E,F). In addition, under DON exposure, the mice showed a more tremendous increase (*p <* 0.05) in the serum concentration of the pro-inflammatory cytokines IL-1β and TNF-α relative to those of the control group (Figure 4G,H). In contrast, the fullerene group showed indices comparable to those of the non-DON control group, with alleviated inflammatory cytokines.

### 3.5. Expression of Inflammatory and Immune Genes

**Figure 5:**
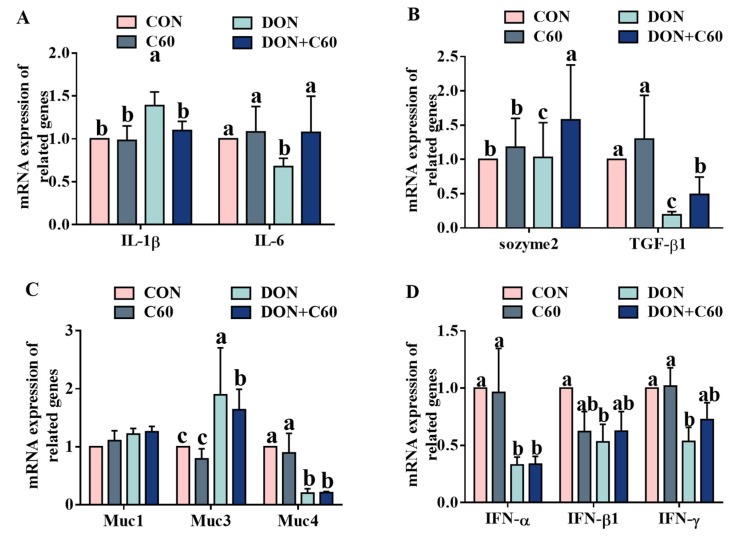
Expression of inflammatory factors and immune gene. (**A**) Expression of *IL-1β* and *IL-6* genes. (**B**) Expression of *sozyme2* and *TGF-β1* genes. (**C**) Expression of *Muc1*, *Muc3*, and *Muc4* genes. (**D**) Expression of *IFN-α*, *IFN-β*, and *IFN-γ* genes. The statistical assay used one-way ANOVA. Data are expressed as means ± SD (*n* = 8), four models chosen; ^a,b,c^ values within a row with different superscripts differ significantly (*p* < 0.05).

Consistent with the concentrations of serum inflammatory factors, compared with those of the non-DON control group (Figure 5), the mice exposed to DON showed higher expression levels of *IL-1β* and *Muc3* in the jejunum, and lower levels of immune-related genes, including that of *TGF-β*, *Muc4*, *IFN-α*, *IFN-β*, and *IFN-γ* (*p <* 0.05). In contrast, fullerene C60 significantly increased (*p <* 0.05) the expression levels of *IL-6*, and *TGF-β* genes in the jejunum, relative to those of the DON group.

### 3.6. Antioxidant Index in Tissue

**Figure 6:**
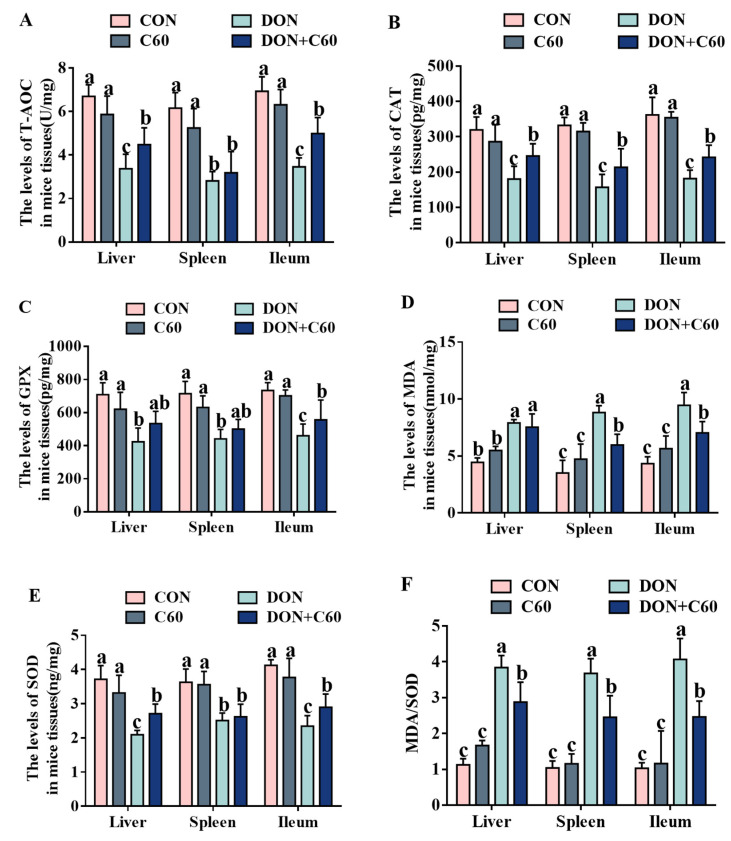
The antioxidant index in tissue. (**A**) The level of total oxidation resistance (T-AOC) in mice tissues, U/mg. (**B**) The level of catalase (CAT) in mice tissues, pg/mg. (**C**) The level of glutathione peroxidase (GPX) in mice tissues, pg/mg. (**D**) The level of malondialdehyde (MDA) in mice tissues, nmol/mg. (**E**) The level of superoxide (SOD) in mice tissues, ng/mg. (**F**) The ratio of MDA/SOD in mice tissues. The statistical assay used one-way ANOVA. Data are expressed as means ± SD (*n* = 8), four models chosen; ^a,b,c^ values within a row with different superscripts differ significantly (*p* < 0.05).

In mice exposed to DON, lower levels of antioxidants were observed in the tissue specimens, with remarkably downregulated concentrations of total antioxidant capacity (T-AOC), catalase (CAT), glutathione peroxidase (GPx), and superoxide dismutase (SOD), as well as upregulation of malondialdehyde (MDA) and the ratio of MDA/SOD compared to those in the control group (*p <* 0.05). In contrast, the addition of fullerene C60 significantly improved antioxidant ability, with higher concentrations of CAT, GPx, SOD, and T-AOC relative to those of the DON group (Figure 6, *p <* 0.05).

### 3.7. Intestinal Morphology and Permeability

**Figure 7:**
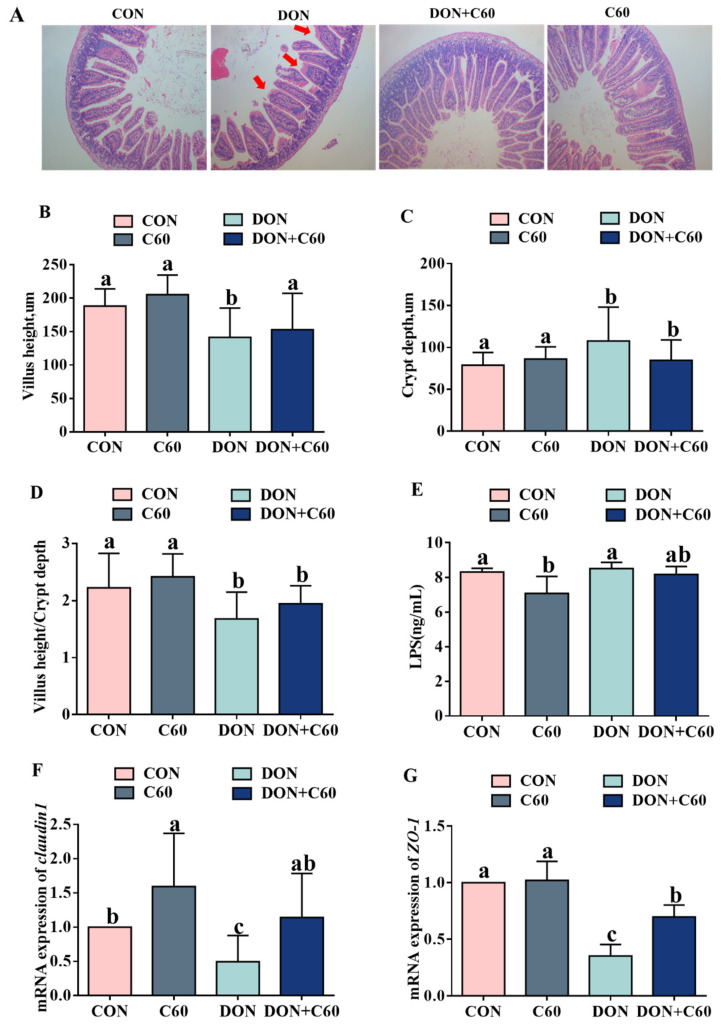
Intestinal epithelial morphology. (**A**) DON resulted in disruption of intestinal morphology. H&E staining of both proximal small intestine from control, DON-treated, DON, and C60-treated mice at d14. (**B**) The height of the villi in the small intestine, μm. (**C**) Crypt depth of small intestine, μm. (**D**) The ratio of villi height to crypt depth in the small intestine. (**E**) Permeability index (LPS) in serum. (**F**) mRNA expression of *claudin 1* genes. (**G**) mRNA expression of *ZO-1* genes. Statistical assay used one-way ANOVA. (*n* = 8), four models are chosen; ^a,b,c^ values within a row with different superscripts differ significantly (*p* < 0.05).

DON treatment disrupted intestinal epithelial morphology, with lower (*p =* 0.049) villus height in the jejunum and higher (*p =* 0.048) crypt depth than those of control mice. In contrast, fullerene C60 significantly improved villus height in DON-treated mice (*p =* 0.040), indicating amelioration of DON-induced injury to the jejunal villi (Figure 7A–D). 

The addition of fullerene C60 decreased the serum lipopolysaccharide LPS concentration compared to the control (*p =* 0.010), and there was a trend to decreased (*p =* 0.052) serum LPS concentration relative to that in the DON-treated group (Figure 7E). 

Intestinal permeability is associated with the levels of tight junction proteins. In agreement with the intestinal morphologic parameters results, compared with the non-DON control, the mRNA expression of *claudin1*, *zonula occludens-1 (ZO-1)* in the jejunum was significantly (*p <* 0.05) downregulated in the mice exposed to DON. Dietary supplementation of fullerene C60 significantly ameliorated the tight-junction expression to levels similar to those in the control group (Figure 7F,G).

## 4. Discussion

DON has been recognized as one of the most common food- and feed-associated mycotoxins. It can produce reactive oxygen species to induce lipid peroxidation and reduce antioxidant enzyme activity, eventually leading to cell injury and death [17,18,28,29], and it has also been reported to activate inflammatory signaling pathways through MAPK, JAK/STAT, and NFκ-B, resulting in apoptosis [30,31]. Due to its high incidence of contamination and occurrence in agricultural commodities with high concentrations, DON poses serious threats to human and animal health, leading to substantial economic losses globally. Therefore, it is urgently needed to find a promising method for preventing its adverse effects. At present, some microorganisms and enzymes have been reported to be able to absorb or degrade mycotoxins and can be used as a potential feed ingredient to counteract the harmful effects of DON in animals [32].

In this study, we investigate the potential of fullerenes to provide protection against DON-induced damage to the growth performance, immune response, and intestine. Although not showing the ability to absorb and degrade mycotoxins, fullerene C60 has documented remarkable biological effects involving oxidative stress, immunomodulation, and autophagy modulation [1,9]. We first evaluated its role in anti-oxidative stress through intestinal cell lines under DON treatment. The addition of fullerene C60 did not degrade the concentration of DON (data not shown), but it substantially decreased cellular ROS levels, improving cell viability. ROS formation can induce oxidative stress, leading to cell damage, and cells have antioxidant networks to scavenge excessively produced ROS. We then investigated whether the supplement with fullerene C60 exhibited better oxidative homeostasis than the control group owing to lower oxidative stress. The RNA-seq showed that two genes, DUOX1 and PXDN, which belong to antioxidant enzymes and have the capacity to scavenge free radicals, have been found to be regulated with supplemented fullerene C60, and the results of cellular antioxidant enzyme activity were consistent with the gene expression trend. These results suggest that fullerene C60 may be used as effective scavengers for ROS in responses to DON-induced cytotoxic effects, attenuating oxidative-stress-induced apoptosis. 

Furthermore, to investigate the role of fullerene C60 on anti-oxidative stress, the immune system, and inflammation in vivo, we analyzed the indices of immune response and inflammation, as well as antioxidant markers of blood and tissue, using a mouse model. DON caused immune suppression, not only decreasing the serum concentrations of IgM and IgG but also significantly interfering with secretory IgA function in the intestinal mucosa. The addition of fullerene C60 helped restore immune function in mice to a level similar to that of the non-DON group. Consistent with immune function, mice with fullerene C60 exhibited milder oxidative stress and proinflammatory status compared to the DON-treated mice. The organ and intestinal morphological analysis also proved that fullerene C60 had protective effects in the liver, spleen, and intestine, and boosted the body to repair oxidative damage, improving the intestinal index. Our results demonstrated that, apart from the clearance of free radicals and reduction in oxidative stress by increasing the level of an endogenous antioxidant enzyme, supplemented fullerene C60 restored the immune system function and alleviated the inflammatory damage. Recent studies also showed that fullerene can induce dendritic cells to become functionally mature and further activate allogeneic T cells, and mice in the presence of fullerene exhibit an enhanced specific Th1-polarized immune response, increasing production of IFN gamma, IL-1beta, and IL-2 [6]. Moreover, the fullerene C60 showed the ability to induce autophagy at low and noncytotoxic concentrations [9], which have been considered to protect intestinal epithelial cells against mycotoxins by alleviating oxidative stress [33]. Thus, combined with the in vivo results, our data indicated that, compared with biological detoxification of DON by microorganisms or enzymes, supplemented fullerene C60 can significantly improve the adverse effect caused by DON on the intestinal cell and immune system by other mechanisms, such as clearance of free radicals and strengthening the immune system function. 

Despite the great progression of dealing with mycotoxins using microbes or enzymes to adsorb or degrade DON, toxin contamination cannot be avoided completely. Thus, it is very critical to research other types of feed additives, and collaborate with each other to provide better protection against DON-induced damage to animal and human health.

## 5. Conclusions

In conclusion, the current study demonstrates that fullerene C60 can improve immune function and intestinal injury by decreasing cellular ROS levels and alleviating oxidative stress. Moreover, under the treatment without DON, supplementation with fullerene C60 showed improved growth performance and immune function, indicating beneficial effects on mouse growth, suggesting its potential as an antidote for DON-induced mycotoxicosis.

## Figures and Tables

**Table 1:** Primers used for real-time quantitative PCR.

Genes	Accession No.	Primers	Sequences (5′-3′)
*β-actin*	NM_007393.3	Forward	GTCCACCTTCCAGCAGATGT
		Reverse	GAAAGGGTGTAAAACGCAGC
*A-chain*	XM_003085465.1	Forward	CGTCCAAGAATTGGATATGA
		Reverse	AGTGACAGGCTGGGATGG
*J-chain*	NM_152839.3	Forward	GAACTTTGTATACCATTTGTCAGACG
		Reverse	CTGGGTGGCAGTAACAACCT
*IL-1β*	NM_008361.3	Forward	ATGAAAGACGGCACACCCAC
		Reverse	GCTTGTGCTCTGCTTGTGAG
*IL-6*	NM_001314054.1	Forward	GCCTTCTTGGGACTGATGCT
		Reverse	TGTGACTCCAGCTTATCTCTTGG
*lysozyme 2*	NM_017372.3	Forward	GAATGGAATGGCTGGCTACT
		Reverse	CGTGCTGAGCTAAACACACC
*TGF-β1*	NM_011577.2	Forward	ACTGGAGTTGTACGGCAGTG
		Reverse	GGATCCACTTCCAACCCAGG
*Muc1*	NM_013605.2	Forward	GTAGGAGCAAGTCACCCCAC
		Reverse	GTTGAGGCGCTTGACAAAGG
*Muc3*	NM_010843.1	Forward	GGCTTTCATCCTCCACTCCC
		Reverse	TTGTGGTGGATGGGGAACTG
*Muc4*	NM_080457.3	Forward	TGTCATTCCACACTCCCAGA
		Reverse	CTAGTCCTACTCTTTGCCCT
*Crs1c*	NM_007844.2	Forward	AAGCGGCAACCAAATCTATG
		Reverse	CCCGAGAGAGCAATACAGGT
*IL-17*	NM_010552.3	Forward	TACCTCAACCGTTCCACGTC
		Reverse	TTTCCCTCCGCATTGACAC
*IFN-α*	NM_010502.2	Forward	TGCCCAGCAGATCAAGAAGG
		Reverse	TCAGGGGAAATTCCTGCACC
*IFN-β1*	NM_010510.1	Forward	CGTGGGAGATGTCCTCAACT
		Reverse	AGATCTCTGCTCGGACCACC

## Data Availability

All data during the study are available from the corresponding author by request.

## Abbreviations

IFN: Interferon; IL, Interleukin; TGF-β, Transforming growth factor-β; TNF-α, Tumor necrosis factor-α; DON, Deoxynivalenol; *ZO-1*, *Zonula occludens-1*; G/F, Gain/feed ratio; T-AOC, Total antioxidant capacity; CAT, Catalase; GPx, Glutathione peroxidase; SOD, Superoxide dismutase; MDA, Malondialdehyde; Muc, mucins; IgG, Immunoglobulin G; IgM, Immunoglobulin M.
